# Induction of H2AX phosphorylation in tumor cells by gossypol acetic acid is mediated by phosphatidylinositol 3-kinase (PI3K) family

**DOI:** 10.1186/s12935-014-0141-5

**Published:** 2014-12-16

**Authors:** Zhong Guo, Jin Zhao, Lei Song, Jian-Xiu Ma, Chen-Jing Wang, Shu-Yan Pei, Chao Jiang, Shang-Biao Li

**Affiliations:** Medical College of Northwest University for Nationalities, Lanzhou, 730030 PR China

**Keywords:** Gossypol acetic acid, H2AX phosphorylation, DNA-PK, ATM, ATR

## Abstract

**Background:**

H2AX is phosphorylated (γH2AX) by members of the phosphatidylinositol 3-kinase (PI3K) family, including Ataxia telangiectasia-mutated (ATM), ATM- and Rad3-related (ATR) and DNA-PK in response to DNA damage. Our study shows that gossypol acetic acid (GAA) alone can induce γH2AX in Human mucoepidermoid carcinoma cell line (MEC-1) in vitro. Thus, we further examined the possible mechanisms of GAA to induce γH2AX in tumor cells.

**Materials and methods:**

The PI3K inhibitors caffeine and wortmannin were used in an effort to identify the kinase(s) responsible for GAA -induced γH2AX in MEC-1 cells. DNA dependent protein kinase (DNA-PK) - proficient and –deficient cells, human glioma cell lines M059K and M059J, were also used to evaluate the kinases responsible for GAA induced H2AX phosphorylation. γH2AX expression was detected by immunofluorescent microscopy. Flow cytometry assay was used to assay γH2AX and cell cycle.

**Results:**

GAA induced H2AX phosphorylation in a cell cycle-dependent manner and a significant G0/G1 phase arrest in MEC-1 cells was shown. Caffeine and wortmannin significantly inhibited GAA-induced H2AX phosphorylation in MEC-1 cells. GAA induced H2AX phosphorylation in M059K, but not in M059J. Taken together, these data suggested that GAA treatment alone could induce H2AX phosphorylation in a cell cycle dependent manner in MEC-1 and M059K, but not in M059J cells. A significant G0/G1 phase arrest was shown in MEC-1.

**Conclusions:**

The member of PI3K family, DNA-PK, ATM and ATR are involved in the H2AX phosphorylation of MEC-1 cells.

## Introduction

Gossypol is a naturally occurring polyphenolic pigment present in cottonseed and in cotton plant byproducts, such as cottonseed oil and cottonseed meal flour. This small molecule, which is well tolerated, exhibits anti-proliferative as well as antimetastatic effects and is also described as a specific inhibitor of the anti-apoptotic proteins Bcl-XL and Bcl-2, thereby inducing apoptosis [1]. Gossypol was also found to increase the radiosensitivity of PC3 cells, contributing to an enhanced rate of apoptosis [2].

Among the many forms of DNA damage, DNA double strand breaks (DSB) are considered the most serious threat to the cell. When not repaired or misrepaired, a single DSB can be lethal. If they do not kill the cell, these breaks can result in mutations, chromosomal rearrangements and compromised genomic integrity [3]. Recently, the relationship between DSB and the histone variant H2AX has attracted much attention. It has been shown that upon DSB generation, the H2AX protein is phosphorylated (termed γH2AX) at serine residue 139 and forms localized “foci” at DSB sites. These foci can then recruit numerous other repair or checkpoint proteins to the damaged sites, including BRCA1, 53BP1, Werner Syndrome protein (WRN), and others [4]. Due to its close relationship with DSB, γH2AX foci formation has been suggested by our laboratory and others as a sensitive method to detect DNA damage [5,6].

The kinases responsible for H2AX phosphorylation have also been studied in detail. It is now clear that members of the phosphatidylinositol 3-kinase family (PI3K), including ATM (ataxia telangiectasia mutated), ATR (ATM and Rad3-related), and DNA-PK (DNA-dependent protein kinase), are involved in the phosphorylation of H2AX, although their roles may differ under different genotoxic stress or in different cell types [7]. For this reason, various kinase inhibitors have been used to differentiate between the involvements of the individual PI3K family members in specific situations. Caffeine is one such inhibitor, and it has been shown that ATM is inhibited by caffeine with an IC50 of 0.2 *m*mol/l, ATR is inhibited with an IC50 of 1.1 *m*mol/l, while DNA-PK is relatively resistant to caffeine with an IC50 of greater than 10 *m*mol/l [8]. Therefore, caffeine is widely used to inhibit ATM and ATR activation. On the other hand, the microbial product wortmannin is a relatively potent inhibitor of DNA-PK (IC50:16 *n*mol/l) and ATM (IC50:150 *n*mol/l) activities, whereas ATR activity is less sensitive to this drug(IC50:1.8 *μ*mol/l) [9].

Recently, gossypol was reported to interfere with inositol phosphate metabolism by inhibiting the activity of inositol-1,4,5-triphosphate 3-kinase (IP3K) isoforms, which are essential for the formation of higher phosphorylated inositols. The level of one isoform of this kinase (IP3K-A) is enhanced in many human tumor cell lines and appears to be associated with a malignant phenotype. One important product of the IP3K dependent metabolism is InsP6. This inositol phosphate is known to be involved in chromatin remodeling and DSB repair processes such as Non-homologous end-joining (NHEJ), as it was shown to interact directly with the Ku70/Ku80 heterodimer, leading to a stimulation of NHEJ [10,11]. These data suggest that a disturbance of the inositol phosphate metabolism by gossypol might also have an effect on DSB repair. Therefore, it was of interest to examine gossypol-triggered induction of γH2AX in tumor cells.

In our previous study, DBS and γH2AX foci have been shown by neutral comet and immunostainning assay in GAA induced MEC-1 cells [12]. Thus, we further examined the possible mechanisms of GAA to induce γH2AX in different tumor cells. By using caffeine and wortmannin, as well as DNA dependent protein kinase (DNA-PK) –proficient and-deficient cells, Human glioma cell line M059K and M059J, the kinases responsible for H2AX phosphorylation induced by GAA were investigated. As reported here, GAA treatment alone could induce H2AX phosphorylation in a cell cycle-dependent manner in MEC-1 and M059K, but not in M059J cells. Also G0/G1 phase arrest is shown MEC-1 cells. The member of PI3K family, DNA-PK, ATM and ATR are involved in the H2AX phosphorylation of MEC-1 cells.

## Materials and methods

### Chemicals and antibodies

GAA, caffeine, wortmannin, 4,6-diamidino-2-phenylindole (DAPI), Propidium Iodide(PI) and dimethysulfoxide (DMSO) were purchased from Sigma (St Louis, MO). Gossypol and wortmannin were dissolved in DMSO and stored at −80°C. Caffeine was dissolved in PBS and stored at −20°C. Anti-γH2AX monoclonal antibody was purchased from Upstate Technology (Lake Placid, NY). Alexa Fluor 488-conjugated AffiniPure Goat Anti-Mouse IgG (H + L) were purchased from Jackson ImmunoResearch (West Grove, PA).

#### Cell culture and treatment

Human mucoepidermoid carcinoma MEC-1 cell was purchased from School of Stomatology Fourth Military Medical University (Xi’an, Shanxi, PRC). DNA dependent protein kinase (DNA-PK) –proficient and -deficient cells, the two human malignant glioma cell lines M059K and M059J, isolated and established by Allalunis-Turner et al. [13], were kept in liquid nitrogen at the Institution of Modern Physics of the Chinese Academy of Science. The cells were maintained as monolayer cultures in Dulbecco’s Modified Eagle’s Medium (DMEM, Invitrogen, Scotland, UK) for MEC-1 and DMEM/F12 (1:1) medium supplemented (10% newborn calf serum, 100 U/ml penicillin, 125 *μ*g/ml streptomycin, and 0.03% glutamine) (Life Technology, Paisley, UK) for M059J and M059K cells. For GAA treatment, various concentrations of GAA were added directly to the appropriate culture plates. Caffeine (8 *μ*mol/l) or wortmannin (20 *n*mol/l) were added 1 h before GAA treatment to inhibit the function of ATM, ATR and DNA-PK.

#### Immunofluorescence microscopy for γH2AX

Immunofluorescent microscopy was conducted according to previously reported procedures with modifications [5]. Briefly, 2 × 10^4^ cells were seeded into 35 mm dishes containing a glass cover slip in each well. After irradiation, slides were air-dried, and fixed for 0.5 h in 2% paraformaldehyde in TBS. Cells were rinsed in TBS, placed in −20°C methanol for 1 min, rinsed, then placed for 20 min in TBS plus 1% bovine serum albumin and 0.2% Tween-20 (TTN) and finally incubated for 2 h with diluted antiphosphohistone H2AX (Ser-139) mAb (Upstate, Lake Placid, NY) diluted 1:500 in TTN. Slides were washed and incubated with FITC-conjugated anti-mouse goat F (ab’) ^2^ fragment (DAKO, Carpinteria, CA) diluted 1:200 in TTN for 1 h at room temperature. Slides were rinsed and then immersed in 0.05 mg/ml DAPI for 15 min, rinsed and mounted with cover slips using 10 *μ*l Fluorogard (Bio-Rad) as the antifade mounting medium, and sealed. To prevent bias in selection of cells that display foci, over 800 randomly selected cells were counted. Cells with three or more foci of any size were classified as positive. All experiments were repeated in triplicate.

#### Flow cytometry assay for γH2AX and cell cycle

Flow cytometry analysis was conducted as described earlier [14,15]. After the various treatments, cells were fixed with cold 70% methanol and kept at −20°C for up to 2 weeks until further analysis. Cells were centrifuged and rinsed with PBS, blocked with PST (4% fetal bovine serum in PBS) for 15 min at room temperature and rinsed with PBS. Cells were incubated with anti-γH2AX monoclonal antibody at a 300-fold dilution for 2 h at room temperature, rinsed with PBS, incubated with Alexa Fluor 488-conjugated AffiniPure Goat Anti-Mouse IgG (H + L) at a 100-fold dilution for another 1 h at room temperature and rinsed again in PBS. Cells were further incubated for 0.5 h at room temperature with 50 *μ*g/ml PI. Filter the sample through a cell strainer (40 μm pore size) and collect single cancer cells samples were analyzed using a flow cytometer (Becton-Dickinson, Bedford, MA, USA). Cell cycle analysis was conducted as described by Amrein et al. [16].

To examine the relationship between GAA-induced γH2AX and cell cycle, the changes in γH2AX immunofluorescence intensity (H2AX IF) were calculated in each phase of the cycle by gating G1, S and G2M cells based on differences in DNA content. The means of γH2AX positive ratios for G1, S and G2/M populations of cells in the DMSO control groups were subtracted from the respective means of the GAA-treated cells. After this subtraction, the GAA-induced changes in positive γH2AX ratio were obtained. Data is presented as the mean of the γH2AX positive ratio of each cell cycle compartment. All experiments were performed three times.

#### Statistical analysis

SPSS version 13.0 software (SPSS Inc., Chicago, Illinois, USA) was used for the statistical analysis. Data are expressed as mean ± standard deviation (SD). A two-tailed Student’s t-test was performed to assess the differences between two groups. Statistical inferences were based on two-sided tests at a significance level of p < 0.05.

## Results

### GAA induces H2AX phosphorylation in a cell cycle-dependent manner in MEC-1 cells

**Figure 1:**
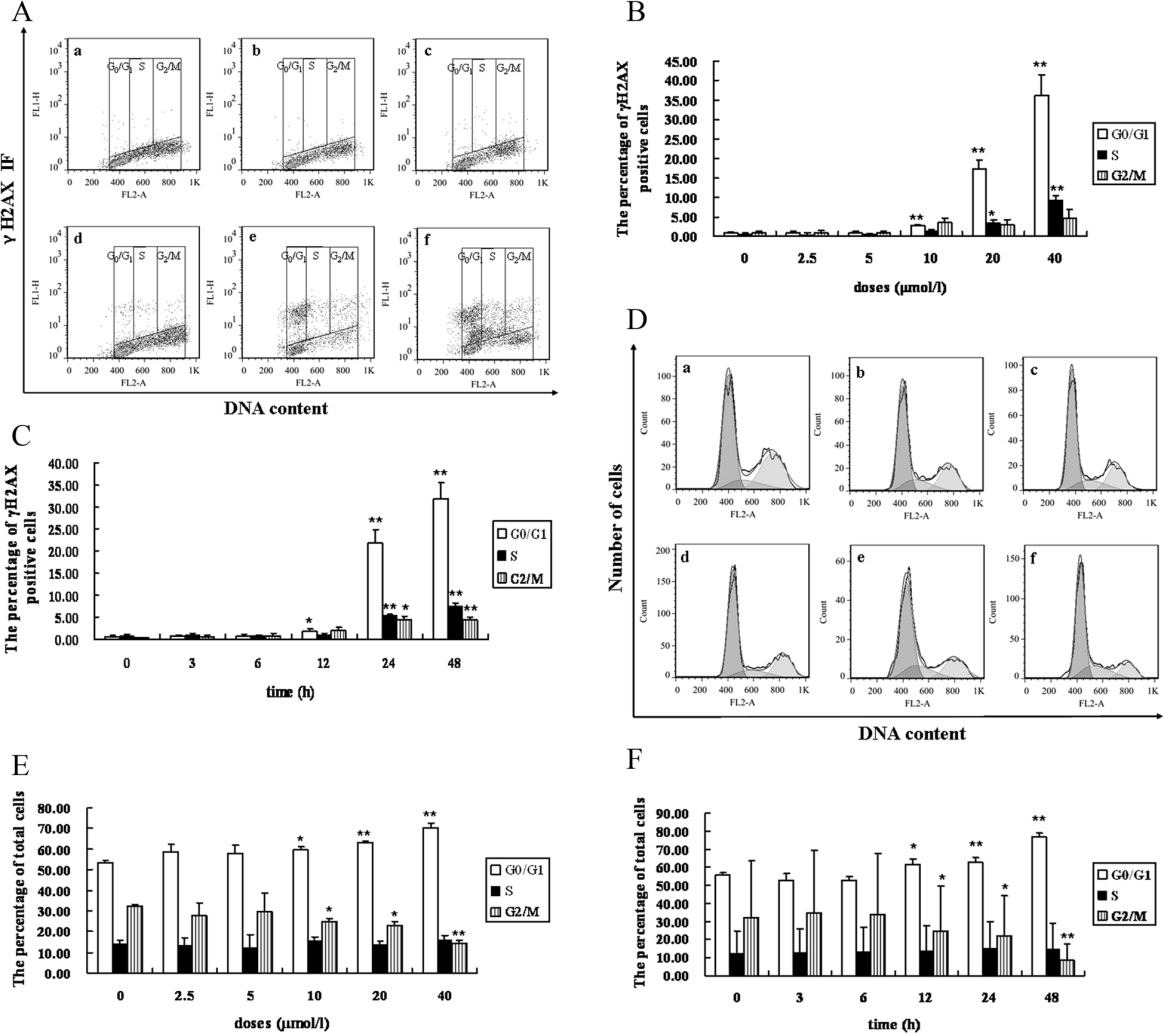
**GAA induces H2AX phosphorylation in a cell cycle-dependent manner in MEC-1 cells. (A)** Bivariate (γH2AX IF vs DNA content) distributions of control and GAA-treated cells (GAA treated for 24 h). **(B)** The percentage of γH2AX positive cells in G1, S and G2/M cells after MEC-1 cells were treated with different doses of GAA for 24 h and 20 *μ*mol/l GAA treated for different time points **(C). (D)** Cell cycle distribution 24 h following GAA treatment. **(E)** Cell cycle distribution of MEC-1 cells after treatment with different doses GAA for 24 h (a: Control. b: 2.5 *μ*mol/l GAA. c: 5 *μ*mol/l GAA. d: 10 *μ*mol/l GAA. e: 20 *μ*mol/l GAA. f: 40 *μ*mol/l)and 20 *μ*mol/l GAA treated for different time points **(F)** n = 3, *: P < 0.05, **: P < 0.01 vs Control.

Typical flow cytometry histograms of GAA-induced phosphorylation of H2AX and cell cycle distribution in MEC-1 cells are shown in Figure 1A and D The percentage of γH2AX positive cells showed little change in all phases when cells were incubated with 2.5 and 5 *μ*mol/l GAA for 24 h, 10 *μ*mol/l GAA had no effect on S and G2/M phase cells. The percentage of γH2AX positive cells had a significant increase at higher doses (20 and 40 *μ*mol/l GAA). G0/G1 phase cells were most sensitive to DNA damage expressing approximately 13.9% and 26.9% higher levels of γH2AX than S and 14.4% and 31.6% higher levels of γH2AX than G2/M phase cells after being treated with 20 and 40 *μ*mol/l GAA respectively for 24 h. It was appear a linear time-related increase in expression γH2AX, especially when the incubation time increased to 24 and 48 h. It is quite evident that compared to S and G2/M phase cells, more G0/G1 phase cells expressed increased levels of γH2AX following GAA treatment with the increase of incubation time (Figure 1B and C).

The dependency of the cell cycle of the MEC-1 cells to GAA exposure is presented in Figure 1E and F. There was a significant G0/G1 phase arrest. After 24 h incubation, 10, 20 and 40 *μ*mol/l GAA induced 59.7%, 63.2% and 70.2% cells in G0/G1 and 20 *μ*mol/l GAA treatment for 12, 24 and 48 h, there were 61.6%, 63.0% and 76.7% cells in G0/G1.

### Wortmannin and caffeine inhibited GAA-induced γH2AX in MEC-1 cells

**Figure 2:**
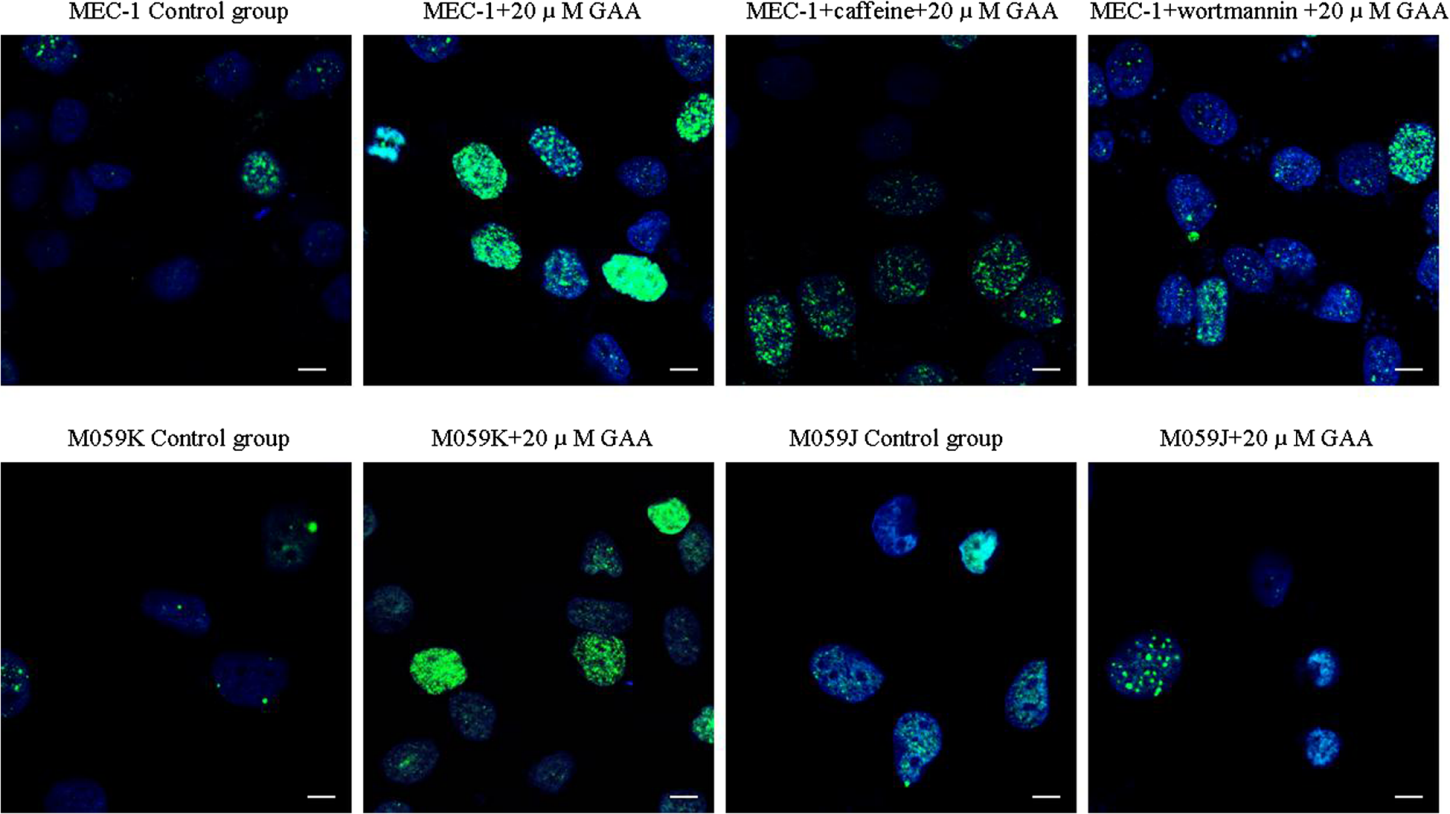
**Digitized images of γH2AX foci in MEC-1 cells, M059K and M059J cells.** MEC-1 cells were pretreated with 8 *m*mol/l caffeine and 20 *n*mol/l wortmannin for 1 h and then were exposed to 20 *μ*mol/l GAA for 24 h. M059K and M059J cells were treated with 20 *μ*mol/l GAA for 24 h. DNA was stained with DAPI and γH2AX was detected using an Alexa 488- conjugated secondary antibody after staining using a monoclonal anti-γH2AX antibody (Scale bar, 15 μm).

**Figure 3:**
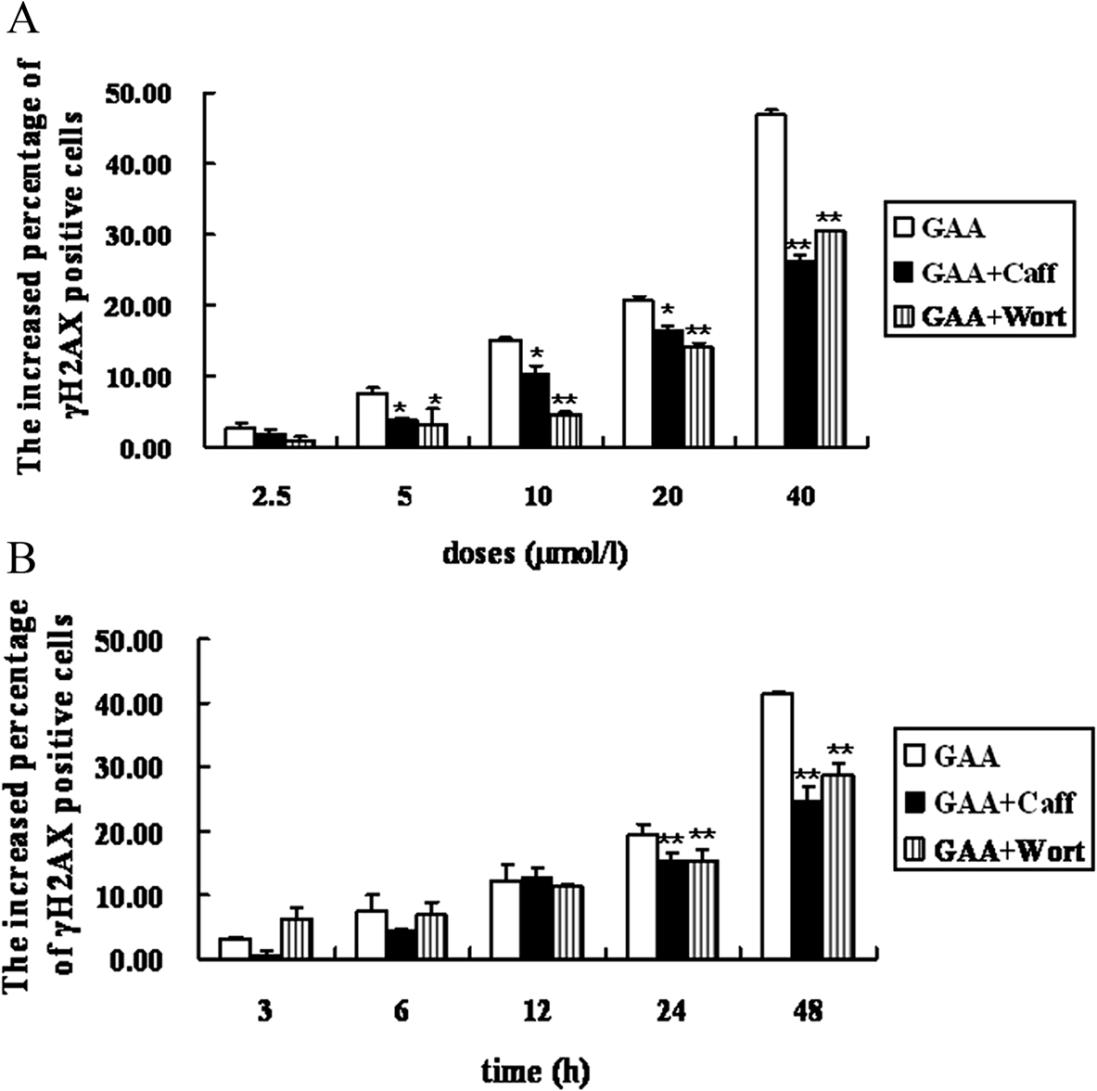
**The dose and time responses of caffeine and wortmannin inhibition of the GAA-induced H2AX phosphorylation in MEC-1 cells.** MEC-1 cells were pretreated with 8 *m*mol/l caffeine and 20 *n*mol/l wortmannin for 1 h and then were exposed to different doses of GAA. γH2AX foci formation was observed by immunofluorescent microscopy. **(A)** Dose responses of GAA-induced H2AX phosphorylation (GAA treated for 24 h); **(B)** Time responses of 20 *μ*mol/l GAA-induced H2AX phosphorylation(. *: P < 0.05, **: P < 0.01 vs GAA treated alone.

Since both flow cytometry and immunofluorescent assays [12] proved GAA could induce the phosphorylation of H2AX in MEC-1 cells, we then were interested in determining which specific PI3K might be responsible for this process. Caffeine, one PI3K inhibitor, should block the action of ATM and ATR, and wortmannin, which should block the action of DNA-PK and ATM. Therefore, MEC-1 cells were preincubated for 1 h with 8 *μ*mol/l caffeine and 20 *n*mol/l wortmannin, a concentration that should inhibit the action of ATM, ATR and DNA-PK. Subsequently the cells underwent GAA treatment [17]. Shown in Figure 2 are representative immunofluorescent images of MEC-1 cells treated with GAA alone, with or without caffeine and wortmannin pre-incubation. The incubation of cells with caffeine and wortmannin alone led to an increase in the level of γH2AX (30.9%, 37.5% and 37.2% in untreated, caffeine and wortmannin treated MEC-1 cells). The increased percentages of γH2AX positive cells were calculated by the γH2AX positive cells percentages of GAA and caffeine (wortmannin) treated groups subtracted from the γH2AX positive cell percentages of GAA treatment alone group. The detailed dose and time response analyses are shown in Figure 3, which illustrates that both caffeine and wortmannin decreased the level of γH2AX in a dose-dependent manner and the inhibitory action of wortmannin was more obvious than caffeine. Caffeine and wortmannin did not decrease the level of γH2AX until 24 to 48 h treatment. Together, these data demonstrate that the member of PI3K family, DNA-PK, ATM and ATR are involved in the H2AX phosphorylation in MEC-1 cells.

### Immunofluorescent assay showed GAA-induced γH2AX in M059K and M059J cells

To further distinguish the kinases responsible for GAA-induced H2AX phosphorylation, DNA dependent protein kinase (DNA-PK) – proficient and -deficient cells, M059K and M059J, were treated with GAA (from 2.5 to 20 *μ*mol/l) at different time points. Figure 2 shows representative immunofluorescent images of M059K and M059J cells treated with 20 *μ*mol/l GAA for 24 h.

**Figure 4:**
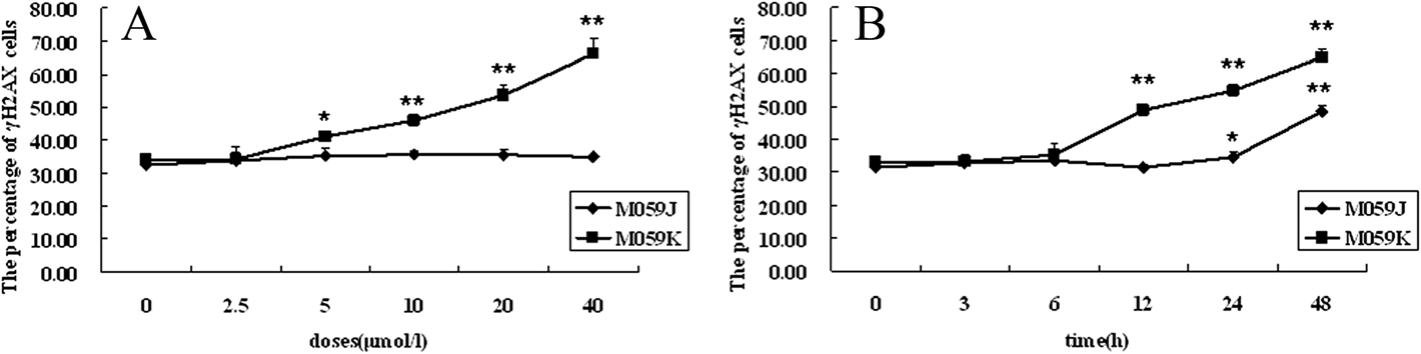
**The dose and time responses of GAA induced H2AX phosphorylation in M059K and M059J cells.** γH2AX foci formation was observed by immunofluorescent microscopy. The dose responses of GAA-induced H2AX phosphorylation in M059K and M059J cells **(A)** (GAA treated for 24 h); The time responses of 20 *μ*mol/l GAA-induced H2AX phosphorylation in M059K and M059J cells **(B)**. *: P < 0.05, **: P < 0.01 vs Control.

Figure 4 depicts the percentage of γH2AX positive cells, clearly demonstrating that GAA could indeed induce γH2AX in a dose- and time-dependent manner in M059K cells, however, for M059J cells, the levels of γH2AX did not increased with the GAA dose, the increase only could be seen at 20 *μ*mol/l GAA incubation for 24 and 48 h. These data further demonstrate that DNA-PK is involved in GAA-induced H2AX phosphorylation in M059K and M059J cells.

### GAA induces γH2AX phosphorylation in a cell cycle-dependent manner in M059K and M059J cells

**Figure 5:**
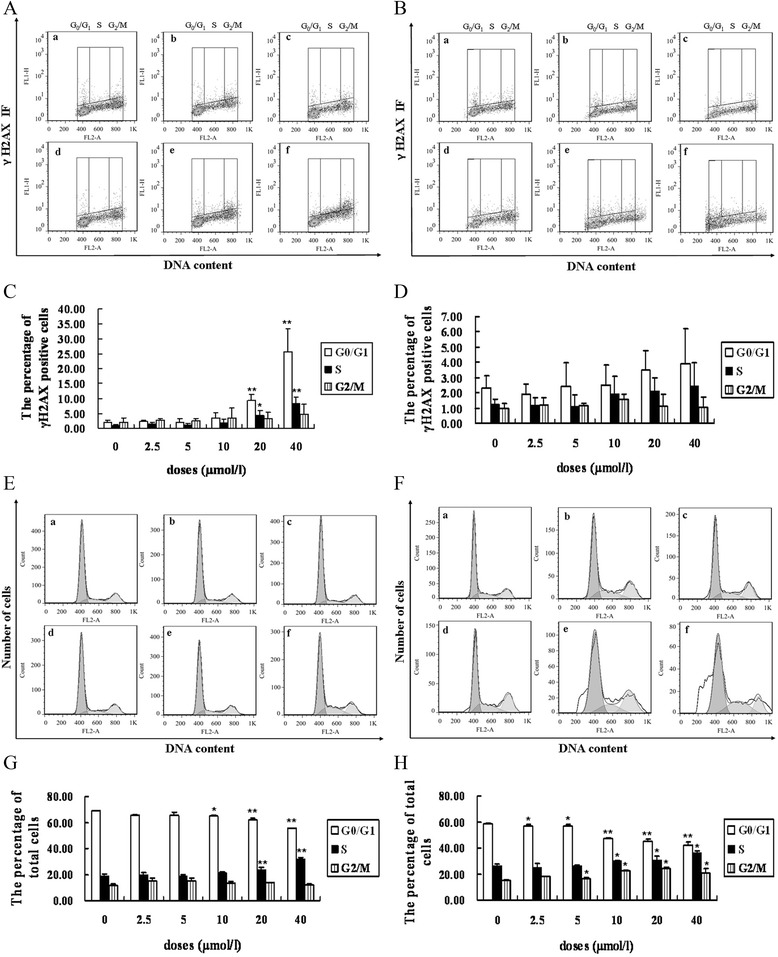
**GAA induced H2AX phosphorylation in a cell cycle-dependent manner in M059K and M059J cells. (A) (B)** Bivariate(γH2AX IF vs DNA content) distributions of control and GAA-treated M059K and M059J cells (GAA treated for 24 h). **(C) (D)** The percentage of γH2AX positive cells in G1, S and G2/M cells after M059K and M059J cells were treated with different doses GAA for 24 h. **(E) (F)** Cell cycle distribution of M059K and M059J cells 24 h following GAA treatment. **(G) (H)** Cell cycle distribution of M059K and M059J cells after treatment with different doses of GAA for 24 h. (ACEG: M059K; BDFH: M059J. a: Control. b: 2.5 *μ*mol/l GAA. c: 5 *μ*mol/l GAA. d: 10 *μ*mol/l GAA. e: 20 *μ*mol/l GAA. f: 40 *μ*mol/l), n = 3, *: P < 0.05, **: P < 0.01 vs Control.

Typical flow cytometry histograms of GAA-induced phosphorylation of H2AX and cell cycle distribution in M059K and M059J are shown in Figure 5A,B,E and F. The dose–response for M059K and M059J cells was similar with the results of the immunofluorescent assay. For M059K cells, the percentage of γH2AX positive cells there was little change in all phases when cells were incubated with lower doses of GAA, however, the percentage of γH2AX positive cells had a significant increase at higher doses. After 24 h incubation of 20 and 40 *μ*mol/l GAA, G0/G1 phase cells express approximately 4.9% and 17.1% higher levels of γH2AX than S and 6.0% and 20.7% higher levels of γH2AX than G2/M phase cells. S phase cells express approximately 1.1% and 3.6% higher levels of γH2AX than G2/M phase cells after 20 and 40 *μ*mol/l GAA treated for 24 h. For M059J cells, the percentage of γH2AX positive cells showed little change in all phases even when the GAA doses reached to 40 *μ*mol/l (Figure 5C and D).

The dependency of the cell cycle of M059K and M059J cells to GAA exposure are presented in Figure 5G and H. S phase cells are more sensitive than other cell cycle-phases cells. After 24 h incubation, 20 and 40 *μ*mol/l GAA induced 24.0% and 31.8% M059K cells in S, 10, 20 and 40 *μ*mol/l GAA induced 30.2%, 30.8% and 36.5% cells in S for M059J cells.

## Discussion

A wealth of published evidence supports the conclusion that H2AX is specifically phosphorylated in response to the occurrence of DSB caused by a variety of genotoxic agents [18,19]. Moreover, at least with ionizing radiation, there appears to be a direct linear correlation between the number of γH2AX foci and the number of DSB, which suggests that each γH2AX focus may represent an individual break [20]. Therefore, when results were reported that gossypol might also have an effect on DSB, it was of interest to investigate this issue in more detail.

As reported here, the result of flow cytometry assay shows that GAA treatment at doses ranging from 2.5-40 *μ*mol/l can induce γH2AX in a dose and time-dependent manner in MEC-1 cells. This was in accordance with the results of immunofluorescence assays we had observed [12]. Interestingly, the degree of H2AX phosphorylation across the cell cycle-phase after GAA exposure was different and a significant G0/G1 arrest was visible. G0/G1 phase cells are much more sensitive and expressed higher levels of γH2AX compared to other cell cycle-phase cells following GAA treatment in MEC-1 cells.

What is the possible significance of these data? One reason maybe that G0/G1 phase cells have an increased vulnerability to oxidant damage during DNA replication. These data can also be explained by the possibility that successful repair of DSB depends on timing in the cell cycle [21]. At last, if proliferating cells exposed to GAA experience similar levels of DSB during each phase of the cell cycle but dissimilar repair rates, they may be particularly susceptible to accumulating deleterious DNA defects during that specific phase.

The observation from the study of PI3K inhibitors is that caffeine and wortmannin inhibited GAA-induced γH2AX in a dose but not time-dependent manner in MEC-1 cells. This observation was not surprising, given the reported action of caffeine and wortmannin, inhibition of ATM, ATR and DNA-PK enzymes that are thought to catalyze γH2AX formation [22,23]. We wandered why for caffeine, the inhibition of GAA-induced γH2AX was not so obvious as wortmannin. Cortez has reported that caffeine does not inhibit ATM and ATR in HCT116 cells. The reason for that, as shown by Cortez, is that caffeine can promote the phosphorylation and activation of ATM under some circumstances [24]. Some other possible explanations may also exist. For example, caffeine may delay the normal dephosphorylation pattern [25] and inhibit homology directed repair [26]. Also, if only a portion of ATR or ATM molecules that are required to prevent DNA damage are accessible to caffeine then the remaining, uninhibited molecules could become superactivated [24]. Finally, it is also possible that ATM or ATR did not phosphorylate H2AX at all in HeLa cells. In all, our data suggest that DNA-PK,ATM and ATR are involved in the H2AX phosphorylation of MEC-1 cells.

Since it is clear that DNA-PK participated in the process of phosphorylation of H2AX [27], DNA dependent protein kinase (DNA-PK)- proficient and –deficient cells, human glioma cell line M059K and M059J, were used to observe the effect of GAA-induced H2AX phosphorylation. Interestingly, differences were observed between M059K and M059J cells in terms of the degree of H2AX phosphorylation. In M059K cells, it was found GAA-induced γH2AX in a dose and time-dependent manner, by contrast, GAA almost could not induce the formation of H2AX in M059J, indicating that DNA-PK is actually responsible for GAA-induced H2AX phosphorylation. Perhaps more interesting was the cell cycle-phase differences on GAA-induced γH2AX phosphorylation in different cells. In M059K cells, S phase cells were much more sensitive and expressed higher levels of H2AX compared to other cell cycle-phase cells following GAA treatment. For M059J cells, the expression of H2AX was not different across the cell cycle phases, but S phase cells was also more sensitive than other cell cycle-phases cells. These results were in accordance with others who have reported that after exposure to different DNA double-strand break-inducing agents, different G2/M or S-phase arrests in M059J and M059K cells are seen [27,28].

In summary, in the present study, we have shown that GAA treatment alone can induce H2AX phosphorylation in the cell lines examined, and members of the PI3K family, DNA-PK, ATM and ATR are also involved in the H2AX phosphorylation in MEC-1 cells. In addition, the degree of H2AX phosphorylation across the cell cycle and cell cycle arrest were different for examined cells. The underlying mechanism is worth further investigation.

## Abbreviations

GAA: Gossypol acetic acid
DMEM: Dulbecco’s modified Eagle’s medium
PI: Propidium Iodide
DMSO: Dimethyl sulfoxide
γH2AX: H2AX phosphorylation
ATM: Ataxia telangiectasia-mutated
ATR: ATM and Rad3-related
DNA-PK: DNA dependent protein kinase
DSB: DNA double strand breaks
NHEJ: Non-homologous end-joining
PI3K: Phosphatidylinositol 3-kinase
